# Regioselective carbon–carbon bond formation of 5,5,5-trifluoro-1-phenylpent-3-en-1-yne

**DOI:** 10.3762/bjoc.9.256

**Published:** 2013-10-23

**Authors:** Motoki Naka, Tomoko Kawasaki-Takasuka, Takashi Yamazaki

**Affiliations:** 1 Division of Applied Chemistry, Graduate School of Engineering, Tokyo University of Agriculture and Technology, 2-24-16, Nakamachi, Koganei 184-8588, Japan

**Keywords:** additives, computation, Li···F chelation, deprotonation, electron-withdrawing effect, organo-fluorine

## Abstract

The regioselective carbon–carbon bond formation was studied using 5,5,5-trifluoro-1-phenylpent-3-en-1-yne as a model substrate, and predominant acceptance of electrophiles β to a CF_3_ group as well as a deuterium trap experiment of the lithiated species led to the conclusion that the obtained regioselectivity is kinetically determined for the reactions with electrophiles, under equilibration of the possible two anionic species.

## Introduction

**Scheme 1:**
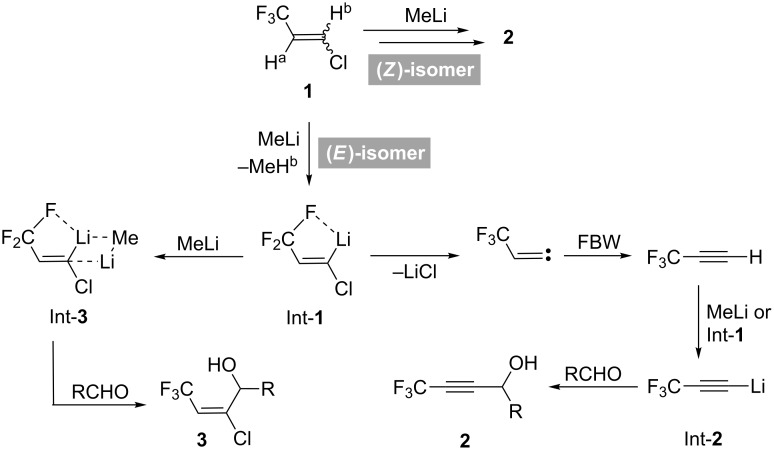
Proposed reaction mechanism between **1** and MeLi.

We have previously reported [1] the interesting behavior of (*E*)-1-chloro-3,3,3-trifluoropropene ((*E*)-**1**) [2–4] towards MeLi, where the proportion of two possible products, propargylic alcohols **2** and allylic alcohols **3**, was proved to be significantly dependent on the equivalents of MeLi used. Thus, as shown in Scheme 1, under the action of up to 1.6 equiv of MeLi, **2** was obtained as a sole product probably by initial H^b^ abstraction from (*E*)-**1** and the resultant Int-**1** was stabilized by the energetically favorable 5-membered intramolecular Li···F chelation [5]. This intermediate Int-**1** experienced Fritsch–Buttenberg-Wiechell (FBW) rearrangement [6–7] to give 3,3,3-trifluoropropyne, and Int-**2** derived from this alkyne eventually captured appropriate aldehydes to afford CF_3_-containing propargylic alcohols **2** [8–12]. Alternatively, stereospecific and exclusive construction of the corresponding allylic alcohols **3** was attained by utilization of greater than 1.7 equiv of MeLi where the stabilized intermediate Int-**3** formed by complexation of Int-**1** with MeLi might play an important role. It turned out that the isomeric (*Z*)-**1** as a substrate furnished only **2** [13] even by the addition of 1.7 equiv of MeLi, presumably as a result of regioselective deprotonation of H^a^, followed by smooth elimination [14–15] of the *trans*-disposed chlorine atom. However, it is also likely that the anionic intermediate produced after H^b^ abstraction would prefer the reaction course to **2** by way of FBW rearrangement because of its lower stability than Int-**1** with loss of the possibility for Li···F chelation.

It was not only MeLi but also LDA which demonstrated this unique base-amount dependent product selectivity of (*E*)-**1** and this substrate was successfully converted to **2** or a mixture of **2**:**3** = 25:75 by the action of 1.2 or 2.2 equiv of LDA, respectively. Although these phenomena are quite interesting, issues remain to be solved for complete mechanistic understanding of the process depicted in Scheme 1.

For clarification of the reactivity of (*E*)-**1** on deprotonation, enyne **4** was used as a model because, after trapping the vinylic anionic species by appropriate electrophiles, comparison of the yields of the resultant regioisomeric **5** and **6** would give us a hint for solving this puzzling question. Moreover, we also expected that such data as well as the additional deuterium trap experiment would offer deeper insight to the actual mechanism.

## Results and Discussion

Investigation of reaction conditions was initially carried out in Et_2_O. A complete recovery of **4** was observed irrespective of the base used (Table 1, entries 1 and 2).

**Table 1:** Investigation of reaction conditions.

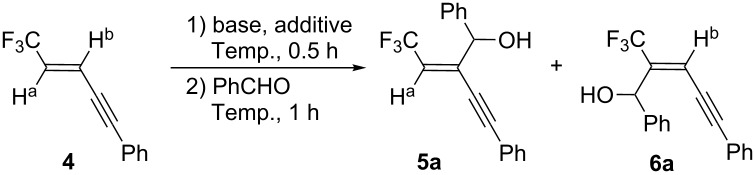							

		Base	Additive	Temp.	^19^F NMR Yield (%)		Recov.^a^
		
Entry	Solvent	(equiv)	(equiv)	(°C)	**5a**	**6a**	(%)

1	Et_2_O	*n*-BuLi (1.0)	—	−80	0	0	100
2		MeLi (1.0)	—	−80	0	0	100
3	THF	*n*-BuLi (1.0)	—	−80	0	0	5
4		MeLi (1.0)	—	−80	0	0	17
5		LDA (1.0)	—	−80	24	6	20
6		PhMgBr (1.0)	—	−80	0	0	17
7		LHMDS (1.0)	—	−80	0	0	17
8		LDA (2.0)	—	−80	41	10	15
9		LDA (3.0)	—	−80	44	8	32
10		LDA (1.0)	HMPA (1.0)	−80	—^b^		
11		LDA (1.0)	DMPU (1.0)	−80	—^b^		
12		LDA (1.0)	TMEDA (1.0)	−80	34	2	11
13		LDA (2.0)	TMEDA (2.0)	−80	68	11	15
14		LDA (3.0)	TMEDA (3.0)	−80	64	19	14
15		LDA (2.0)	TMEDA (2.0)	−100	19	5	76
16		LDA (2.0)	TMEDA (2.0)	−60	50	17	1
17		LDA (2.0)	TMEDA (2.0)	−40	—^b^		43
18		LDA (2.0)	TMEDA (2.0)	−80^c^	54	17	8

^a^Recovered starting material. ^b^Almost no fluorinated products were detected by ^19^F NMR. ^c^After addition of PhCHO, stirring was continued for 1 h at −80 °C, followed by 3 h at 0 °C.

Neither were **5a** and **6a** detected in THF when MeLi or *n*-BuLi were employed and this system produced only a complex mixture (Table 1, entries 3 and 4). A survey of bases established that, in spite of failures with PhMgBr and LHMDS, the use of LDA led to the formation of **5a** and **6a** in 24 and 6% yields, respectively, after trapping the resultant anionic intermediates by PhCHO (Table 1, entries 5 to 7). An increase in LDA concentration to 2 equiv improved the yield of product to 41% (**5a**) and 10% (**6a**), but additional LDA was not effective (Table 1, entries 8 and 9). Subsequent to determining that LDA was the base of choice, a brief study of the effect of additives was performed. The addition of HMPA or DMPU was detrimental and no trace amount of **4**, **5a**, nor **6a** was detected (Table 1, entries 10 and 11). However, TMEDA affected this process to some extent (Table 1, entries 5 vs 12) [16–18], and the addition of 2 equiv each of LDA and TMEDA produced the regioisomeric **5a** and **6a** in better yields of 68% and 11%, respectively (Table 1, entry 13). The recovery of 15% of the substrate **4** under these conditions prompted further raise in their amount to 3 equiv, but again, no significant improvement was noticed (Table 1, entries 13 vs 14). The effect of temperature on the reaction was crucial. Thus, efficient deprotonation was not occurred at −100 °C (Table 1, entry 15) with the consequence that decomposition of the vinylic anions at −40 °C led to formation of different intermediates which would further consume LDA, leaving a larger amount of **4** unreacted (Table 1, entry 17). In conclusion, the reaction conditions in entry 13 in Table 1 were selected to be optimal.

With optimized conditions in hand, the scope and limitation of this procedure were investigated. A number of carbonyl compounds were employed as electrophiles for the anions generated from the enyne **4**. Benzaldehydes with electron-donating (Table 2, entries 2 and 3) as well as -withdrawing (Table 2, entry 4) substituents at the para position were nicely accepted as electrophiles. The allylic alcohols **5** were formed as the major product without exception in a similar **5**/(**5**+**6**) ratio of 70–75% (Table 2, entries 1 to 5). An inseparable mixture of the compounds **5** and **6** was also synthesized from aliphatic aldehydes as shown in entries 5 and 6 in Table 2. Their proportion seemed to be affected by the bulky substituent and pivalaldehyde attained the higher ratio of 88%. Although **5** and **6** were obtained in 50 to 70% combined yields by the reaction with aldehydes, lower reactivity was displayed by the less electrophilic and more hindered acetophenone. Products **5g** and **6g** were formed in 15% and 8% yields, respectively, while there was a 52% recovery of **4** under these conditions detected by ^19^F NMR (Table 2, entry 7).

**Table 2:** Scope and limitation of the present reactions.

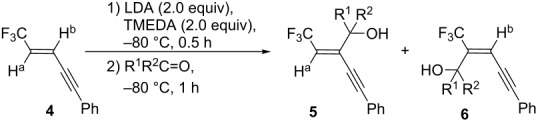							

				Isolated yield^a^ (%)		**5**/(**5**+**6**)^b^	Recov.^c^
Entry	R^1^	R^2^	Product	**5**	**6**	(%)	(%)

1	Ph-	H	**a**	50 (65)	21 (25)	72	15
2	*p*-Me-C_6_H_4_-	H	**b**	49 (52)	17 (17)	75	18
3	*p*-MeO-C_6_H_4_-	H	**c**	49 (43)	15 (17)	72	16
4	*p*-F_3_C-C_6_H_4_-	H	**d**	36 (43)	12 (17)	72	32
5	Et-	H	**e**	47 (57)	16 (19)	75	26
6	*t*-Bu-	H	**f**	54 (59)	10 (8)	88	23
7	Ph-	Me-	**g**	(15)	(8)	65	52

^a^In the parenthesis were shown the yields determined by ^19^F NMR. ^b^These ratios were determined by ^19^F NMR for the crude materials. ^c^Recovered starting material.

**Scheme 2:**
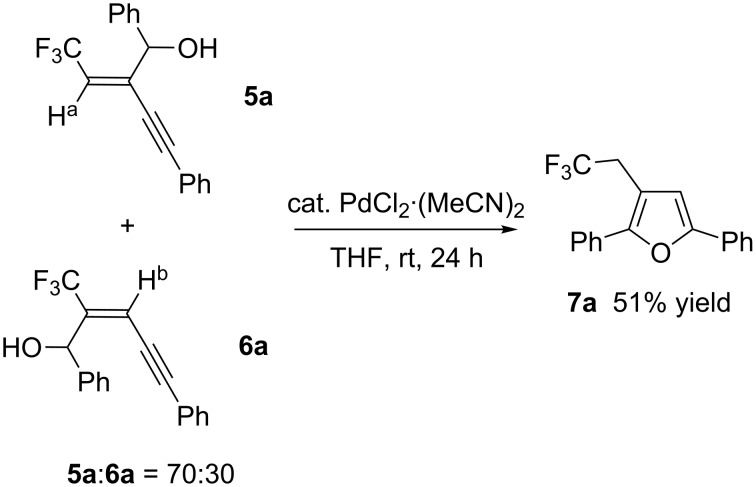
Furan synthesis from a mixture of **5a** and **6a**.

Compounds **5** and **6** were readily characterized by ^19^F NMR spectra. The former material showed clear ^3^*J* couplings of 7 to 9 Hz between F and H^a^, but with the latter ^4^*J*_F-Hb_ was usually not observed. Moreover, the fact that isolation of the furan **7a** in 51% yield was realized by subjection of a 70:30 mixture of inseparable **5a** and **6a** to the already reported Pd-catalyzed cyclization conditions [19] suggested that the major isomer should be **5a**, not **6a** (Scheme 2). In the case of **5** and **6**, electrophiles were incorporated without stereochemical contamination in all instances.

**Scheme 3:**
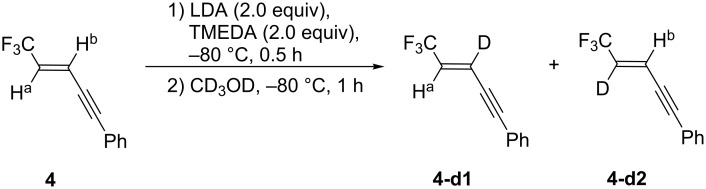
Deuteration of anionic species from **4**.

For obtaining further mechanistic proofs for the present reaction, we have planned to capture the intermediary anionic species with the aid of the usual deuteration technique. Thus, a large excess amount (26 equiv) of CD_3_OD was introduced to a solution containing the anionic species which was prepared from **4** by the standard conditions, which expected us to obtain a mixture of **4-d1** and **4-d2** or either of them predominantly (Scheme 3).

**Figure 1:**
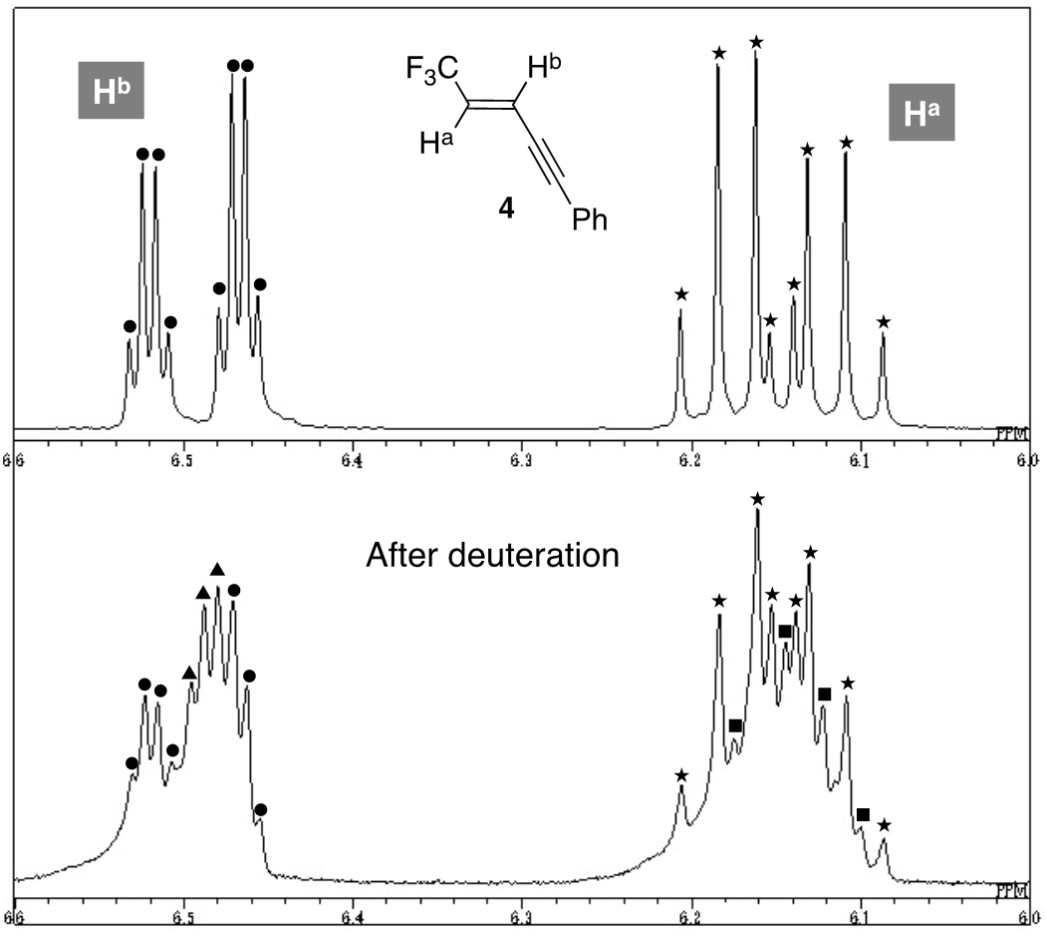
A part of ^1^H NMR chart of **4** and its deuterated mixture.

Observation of the well-resolved two sets of peaks was possible for the two vinylic protons H^a^ and H^b^ in the enyne **4** at δ 6.15 (qd, *J* = 6.6, 15.9 Hz) and 6.48 (qd, *J* = 2.4, 15.9 Hz), respectively (Figure 1 and Supporting Information File 1).

On the other hand, relatively complex peaks were detected from the crude deuterated mixture due to incomplete quench of the reactive anionic species by CD_3_OD, but new resonance peaks (indicated by ■ and▲) undoubtedly appeared with overlapping the ones of the original H^a^ (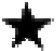) and H^b^ (●). At the lower field (around 6.5 ppm) area, peaks indicated by ▲ are considered as a part of quartet with a coupling constant of 2.3 Hz, which is close to the ^4^*J*_H-F_ coupling value of **4** shown above. Moreover, disappearance of the large ^3^*J*_H-H_ constant typical for (*E*)-alkenes suggested that a deuterium atom should be incorporated at the site where H^a^ was originally situated and that **4-d2** was actually produced. It is also clear that the H^a^ region also contains a couple of peaks (■) with the ^3^*J*_H-F_ coupling constant of 6.6 Hz basically identical to the one of **4**, which anticipated us the simultaneous formation of the regioisomeric **4-d1** on the basis of the similar consideration.

**Scheme 4:**
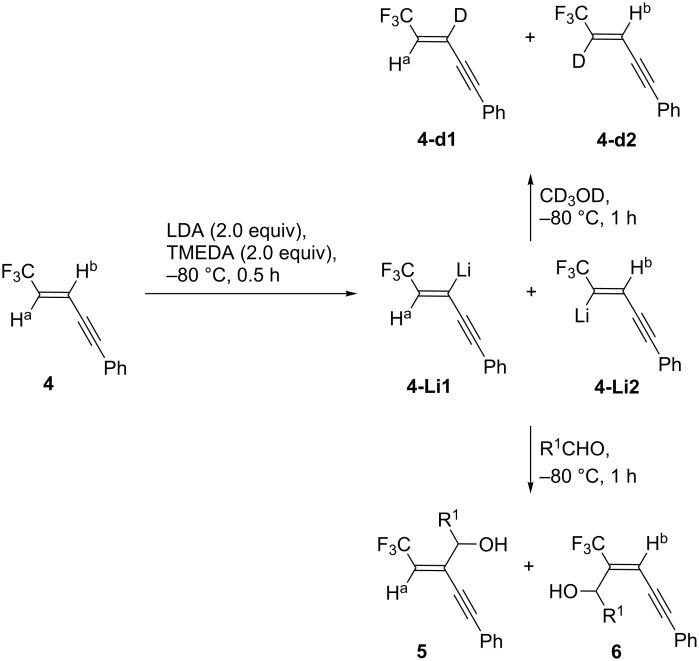
Lithiation of **4** and the following electrophilic reactions.

Thus, lithiation of **4** and the following quench with CD_3_OD furnished a mixture of **4-d1** and **4-d2**, which directly proved that the both lithiated species **4-Li1** and **4-Li2** were actually generated (Scheme 4), although it is quite unfortunate not to be able to quantitatively discuss the proportion of **4-d1** to **4-d2**. On the other hand, as apparent from Table 2, aldehydes preferentially yielded **5** with qualitatively better ratio of **5**/**6** than the one of **4-d1**/**4-d2**. Our computation of **4-Li1** and **4-Li2** by Gaussian 09W [20] using the B3LYP/6-31+G* level of theory uncovered that the former was energetically more favorable than the latter by 5.34 (3.67) kcal/mol under vacuum (in THF [21]), which would indicate that intramolecular Li···F chelation is contributed to the stability more significantly rather than the electron-withdrawing stabilization by the CF_3_ group. Thus, with this computational information in hand, the process shown in Scheme 4 would be elucidated as follows: deuteration would quickly occur to afford **4-d1** and **4-d2** whose proportion would reflect the ratio of **4-Li1** and **4-Li2**. On the other hand, less reactive aldehydes should be captured more slowly and the product preference of **5** to **6** would be kinetically determined by their activation energy difference with equilibration between these two lithiated species in the presence of such a proton source as iPr_2_NH [22].

**Scheme 5:**
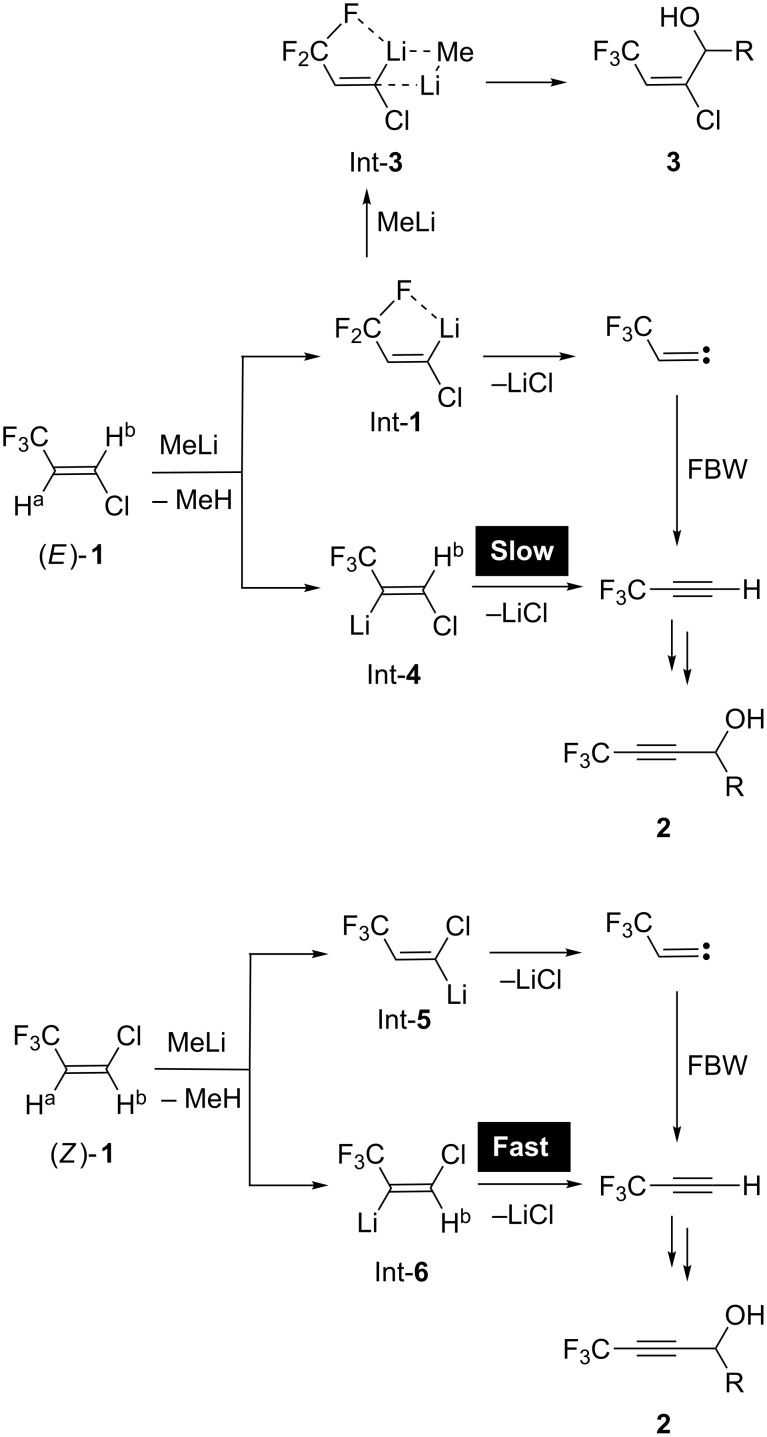
Alternative reaction mechanism between **1** and MeLi.

This interpretation also led to the reconsideration of the proposed mechanism shown in Scheme 1. If deprotonation of both H^a^ and H^b^ in (*E*)-**1** was possible, lithiated species Int-**4** and Int-**1** was obtained, respectively (Scheme 5). Due to slow elimination of a LiCl molecule from Int-**4** due to the *cis* relationship of Li and Cl, Int-**4** would act as a base to abstract a terminal proton of 3,3,3-trifluoropropyne via FBW rearrangement of Int-**1**. The regenerated (*E*)-**1** would be converted to a mixture of Int-**1** and Int-**4** again to eventually yield the propargylic alcohols **2**. However, a larger amount of MeLi would allow constructing the complex Int-**3** presumably with possessing better stability and the allylic alcohols **3** were obtained. In the case of the stereoisomeric (*Z*)-**1**, situation was totally different, and Int-**5** without intramolecular chelation should accelerate the elimination of LiCl and the ideal *trans* disposition of Li and Cl in Int-**6** also favored the route to trifluoropropyne, and as a result, propargylic alcohols **2** were selectively afforded.

## Conclusion

By using the representative model substrate **4**, we have reached to a conclusion that its initial lithiation would occur at both vinylic sites and the following reactions with appropriate electrophiles would proceed under kinetic control where equilibration of the resultant lithiated species might be effective if the capture of electrophiles was slow. This important information allowed us to reconsider our previously proposed mechanism as shown in Scheme 5.

## Supporting Information

File 1 Experimental.
