# Supplementary material for: Regioselective carbon–carbon bond formation of 5,5,5-trifluoro-1-phenylpent-3-en-1-yne
Source: Beilstein J Org Chem. 2013 Oct 23;9:2182–8. doi: 10.3762/bjoc.9.256 (PMC3817480; doi:10.3762/bjoc.9.256)
Supplement: File 1 — Experimental. [file Beilstein_J_Org_Chem-09-2182-s001.pdf]

Supporting Information

for

Regioselective carbon–carbon bond formation of 5,5,5-trifluoro-1-phenylpent-3-en-1-yne

Motoki Naka, Tomoko Kawasaki-Takasuka, and Takashi Yamazaki*

Address: Division of Applied Chemistry, Graduate School of Engineering, Tokyo University of Agriculture and Technology, 2-24-16, Nakamachi, Koganei 184-8588, Japan

Email: Takashi Yamazaki - tyamazak@cc.tuat.ac.jp

* Corresponding author

Experimental

General

Unless otherwise noted, materials were obtained from commercial suppliers and were used without further purification. All manipulations involving air-sensitive materials were performed under argon. Anhydrous Et_2O, THF and CH_2Cl_2 were purchased and were used without further purification.

^{1}H, ^{13}C, and ^{19}F NMR spectra were recorded with a JEOL JNM-LA300 (^{1}H : 300.40 MHz, ^{13}C: 75.45 MHz, and ^{19}F: 282.65 MHz), in $CDCl_3$. Chemical shifts were recorded in parts per million (ppm), downfield from internal tetramethylsilane (for ^{1}H and ^{13}C NMR, Me_4Si : δ 0.00 ppm, and for ^{19}F NMR, C_6F_6: δ –163 ppm). Infrared (IR) spectra were obtained on a JASCO FT/IR-4100 spectrometer, and all spectra were reported in wave numbers (cm^{-1}). Analytical thin-layer chromatography (TLC) on silica gel 60 F_{254} (Merck) was routinely used for monitoring reactions. Column chromatography was conducted with silica gel 60 N (spherical, neutral, 63–210 nm, Kanto). Elemental analyses were performed by Perkin-Elmer SeriesII CHNS/O analyzer.

Due to inseparability of the compounds **5** and **6**, IR and elemental analysis were performed as a mixture, and it is possible that some peaks of the minor isomers **6** were not properly detected by NMR.

(*E*)-5,5,5-Trifluoro-1-phenylpent-3-en-1-yne (4) [1]

To a 30 mL round-bottomed flask containing 2.0 mL (20.0 mmol) of (*E*)-1-chloro-3,3,3-trifluoropropene (*E*)-**1** and THF (10 mL) were successively added 1.461 g (20.0 mmol) of diethylamine**,** 2.20 mL (20.0 mmol) of phenylacetylene**,** 0.1143 g (0.10 mmol) of tetrakis-(triphenylphosphine)palladium and 0.076 g (0.40 mmol) of CuI at 0 °C, and the whole was stirred 24 h at room temperature. After addition of H_2O (100 mL), the resultant mixture was extracted with Et_2O (100 mL) twice and dried over anhydrous Na_2SO_4. Filtration and evaporation furnished a crude mixture which was chromatographed on silica-gel (*n*-hexane:CH_2Cl_2=6:1) to give 3.0419 g (15.488 mmol) of **4** in a stereospecific fashion. Yield 77%. R_f = 0.64 (*n*-hexane:CH_2Cl_2=6:1). ^{1}H NMR δ 6.15 (1H, qd, *J*=6.6, 15.9 Hz), 6.48 (1H, qd, *J*=2.4, 15.9 Hz), 7.31-7.48 (5H, m). ^{13}C NMR δ 84.1, 96.4, 118.9 (q, *J*=269.2 Hz), 121.9, 122.6 (q, *J*=8.1 Hz), 127.2 (q, *J*=34.1 Hz), 128.5, 129.3, 131.9. ^{19}F NMR δ –65.90 (d, *J*=6.8 Hz).

General procedure of anion formation from 3 and its reaction with appropriate electrophiles. (*Z*)-4,4,4-Trifluoro-1-phenyl-2-(2-phenylethynyl)but-2-en-1-ol (5a) and (*E*)-1,5-diphenyl-2-(trifluoromethyl)pent-2-en-4-yn-1-ol (6a)

To a 30 mL round-bottomed flask containing 1.5 mL of THF and 1.0 mmol of LDA (prepared from diisopropylamine (0.1012 g, 1.0 mmol) and BuLi (1.65 *M*, 0.60 mL, 1.0 mmol) at 0 °C) was added tetramethylenediamine (0.1162 g, 1.00 mmol) and the solution was stirred for 5 min. (*E*)-5,5,5-Trifluoro-1-phenylpent-3-en-1-yne **4** (0.0981 g, 0.500 mmol) was added at –80 °C, and after 30 min at the same temperature, benzaldehyde (0.0584 g, 0.550 mmol) was introduced and the mixture was further stirred for 1 h. The reaction was quenched with 1 *M* HCl (5 mL), and the resultant oil was extracted with AcOEt twice and dried over anhydrous $MgSO_4$. Filtration and evaporation furnished a crude materials which was purified by chromatography on silica-gel (AcOEt:*n*-hexane=10:1) to yield 0.1066 g (0.3526 mmol) of the desired compounds as an inseparable mixture (**5a**:**6a**=79:21). Combined yield 71%. R_f =0.38 (*n*-hexane:AcOEt=4:1). IR (neat) ν 3559, 3393, 3064, 3034, 2925, 2850, 2204, 1633, 1492, 1452, 1443, 1336, 1258, 1166, 1122, 1090, 1043, 1027, 918, 895, 859, 834, 756, 719, 699, 689, 671, 634, 610 cm^{-1}. Anal. Calcd for $C_{18}H_{13}F_3O$: C, 71.52; H, 4.33. Found: C, 71.71; H, 4.47.

5a ^{1}H NMR δ 2.47 (1H, d, *J*=6.6 Hz), 5.89 (1H, d, *J*=6.3 Hz), 6.11 (1H, q, *J*=9.0 Hz), 7.28-7.90 (20H, m). ^{13}C NMR δ 70.1 (q, *J*=1.8 Hz), 84.5, 96.6, 115.7 (q, *J*=7.5 Hz), 122.5 (q, *J*=271.0 Hz), 121.5, 122.7 (q, *J*=34.7 Hz), 126.1, 128.2, 128.4, 129.3, 131.8, 139.9. ^{19}F NMR δ –56.91 (d, *J*=9.0 Hz).

6a ^{1}H NMR δ 2.63 (1H, brs), 6.05 (1H, s), 6.51 (1H, s), 7.28-7.90 (20H, m). ^{13}C NMR δ 70.8, 83.0, 102.2, 121.6, 125.7, 127.8, 128.5, 129.5, 131.7, 137.9 (q, *J*=5.6 Hz), 140.3, 141.6 (q, *J*=24.1 Hz). ^{19}F NMR δ –64.05 (s).

(*Z*)-4,4,4-Trifluoro-1-(4-methylphenyl)-2-(2-phenylethynyl)but-2-en-1-ol (5b) and (*E*)-1-(4-methylphenyl)-5-phenyl-2-(trifluoromethyl)pent-2-en-4-yn-1-ol (6b)

Combined yield 66%, R_f =0.45 (*n*-hexane:AcOEt=4:1). IR (neat) ν 3395, 3057, 3033, 2924, 2204, 1632, 1514, 1491, 1443, 1412, 1380, 1338, 1314, 1259, 1165, 1123, 1087, 1043, 994, 917, 896, 863, 847, 806, 781, 757, 729, 689, 630 cm^{-1}. Anal. Calcd for $C_{19}H_{15}F_3O$: C, 72.14; H, 4.78. Found: C, 71.77; H, 4.88.

5b ^{1}H NMR δ 2.35 (1H, s), 2.43 (1H, d, *J*=5.1 Hz), 5.84 (1H, d, *J*=4.2 Hz), 6.07 (1H, q, *J*=8.7 Hz, a), 7.16-7.42 (18H, m). ^{13}C NMR δ 55.2, 69.8 (q, *J*=1.9 Hz), 84.7, 96.5, 121.6, 122.5 (q, *J*=34.7 Hz), 123.4 (q, *J*=271.0 Hz), 127.4, 128.4, 129.3, 131.8, 132.2, 138.1 (q, *J*=5.6 Hz), 159.5. ^{19}F NMR δ –56.90 (d, *J*=9.0 Hz).

6b ^{1}H NMR δ 2.34 (1H, s), 2.60 (1H, d, *J*=5.1 Hz), 6.02 (1H, d, *J*=5.7 Hz), 6.56 (1H, s), 7.16-7.42 (18H, m). ^{13}C NMR δ 70.7, 83.1, 102.0, 115.2 (q, *J*=7.4 Hz), 127.1, 128.5, 129.5, 131.7, 132.5, 159.2. ^{19}F NMR δ –64.03 (s).

(*Z*)-4,4,4-Trifluoro-1-(4-methoxyphenyl)-2-(2-phenylethynyl)but-2-en-1-ol (5c) and (*E*)-1-(4-methoxyphenyl)-5-phenyl-2-(trifluoromethyl)pent-2-en-4-yn-1-ol (6c)

Combined yield 65%, R_f =0.31 (*n*-hexane:AcOEt=4:1). IR (neat) ν 3649, 3431, 3064, 3002, 2956, 2921, 2839, 2204, 1631, 1611, 1587, 1491, 1464, 1443, 1419, 1337, 1304, 1086, 1033, 918, 895, 863, 848, 820, 781, 757, 689, 628 cm^{-1}. Anal. Calcd for $C_{19}H_{15}F_3O_2$: C, 68.67; H, 4.55; Found: C, 69.03; H, 4.71.

5c ^{1}H NMR δ 2.42 (1H, d, *J*=6.3 Hz), 3.80 (3H, s), 5.82 (1H, d, *J*=5.7 Hz), 6.07 (1H, q, *J*=9.0 Hz), 7.29-7.47 (18H, m). ^{13}C NMR δ 69.7 (q, *J*=1.9 Hz), 84.0, 97.2, 121.2, 122.4 (q, *J*=271.0 Hz), 123.4 (q, *J*=35.3 Hz), 124.0 (q, *J*=271.6 Hz), 125.4 (q, *J*=3.7 Hz), 126.5, 128.5, 129.6, 130.4 (q, *J*=32.3 Hz), 131.9, 137.1 (q, *J*=5.6 Hz), 143.7. ^{19}F NMR δ –56.80 (d, *J*=9.0 Hz), –63.80 (s).

6c ^{1}H NMR δ 2.60 (1H, d, *J*=6.3 Hz), 3.81 (3H, s), 6.00 (1H, d, *J*=6.3 Hz), 6.53 (1H, s), 7.29-7.47 (18H, m). ^{13}C NMR δ 70.1, 82.5, 102.6, 116.6 (q, *J*=6.9 Hz), 121.3, 126.0, 128.6, 129.8, 131.8, 144.1 (q, *J*=1.3 Hz). ^{19}F NMR δ –63.78 (s), –63.84 (s).

(*Z*)-4,4,4-Trifluoro-2-(2-phenylethynyl)but-1-[4-(trifluoromethyl)phenyl]-2-en-1-ol (5d) and (*E*)-5-phenyl-2-(trifluoromethyl)-1-[4-(trifluoromethyl)phenyl]pent-2-en-4-yn-1-ol (6d)

Combined yield 48%, R_f =0.44 (*n*-hexane:AcOEt=4:1). IR (neat) ν 3853, 3734, 3676, 3648, 3376, 3067, 2919, 2849, 2204, 1633, 1491, 1444, 1415, 1089, 1017, 897, 857, 819, 757, 688, 673, 609 cm^{-1}. Anal. Calcd for $C_{19}H_{12}F_6O$: C, 61.63; H, 3.27. Found: C, 61.90; H, 3.26.

5d ^{1}H NMR δ 2.60 (1H, d, *J*=6.0 Hz), 5.94 (1H, d, *J*=5.7 Hz), 6.15 (1H, q, *J*=8.7 Hz), 7.32-7.65 (18H, m). ^{13}C NMR δ 26.2, 35.7, 75.2 (q, *J*=1.8 Hz), 86.1, 96.3 (q, *J*=1.3 Hz), 121.8, 122.3 (q, *J*=270.4 Hz), 124.4 (q, *J*=34.7 Hz), 128.5, 129.3, 131.61, 136.4 (q, *J*=5.6 Hz). ^{19}F NMR δ –57.14 (d, *J*=9.0 Hz).

6d ^{1}H NMR δ 2.72 (1H, d, *J*=5.7 Hz), 6.12 (1H, d, *J*=5.7 Hz), 6.60 (1H, s), 7.32-7.65 (18H, m). ^{13}C NMR δ 26.1, 37.7, 78.1, 81.5, 102.3, 117.2 (q, *J*=7.5 Hz), 121.9, 128.6, 129.5, 131.55, 141.6 (q, *J*=26.6 Hz). ^{19}F NMR δ –61.12 (s).

(*Z*)-6,6,6-Trifluoro-4-(2-phenylethynyl)hex-4-en-3-ol (5e) and (*E*)-7-phenyl-4-(trifluoromethyl)hept-4-en-6-yn-3-ol (6e)

Combined yield 63%, R_f =0.41 (*n*-hexane:AcOEt=4:1). IR (neat) ν 3566, 3393, 3062, 2972, 2938, 2881, 2204, 1632, 1491, 1458, 1443, 1352, 1338, 1306, 1070, 1026, 980, 917, 881, 756, 689, 677 cm^{-1}. Anal. Calcd for $C_{14}H_{13}F_3O$: C, 66.14; H, 5.15. Found: C, 65.89; H, 5.19.

5e ^{1}H NMR δ 0.98 (3H, t, *J*=7.4 Hz), 1.70-1.94 (4H, m), 1.92 (1H, d, *J*=7.5 Hz), 4.57-4.64 (1H, m), 6.05 (1H, q, *J*=8.7 Hz), 7.32-7.51 (10H, m). ^{13}C NMR δ 21.10, 70.1 (q, *J*=1.8 Hz), 84.6, 96.5, 121.6, 122.5 (q, *J*=34.7 Hz), 123.4 (q, *J*=271.0 Hz), 126.0, 128.4, 129.1, 129.3, 131.9, 137.1, 138.0, 138.1 (q, *J*=5.6 Hz). ^{19}F NMR δ –57.21 (d, *J*=6.8 Hz).

6e ^{1}H NMR δ 1.06 (3H, t, *J*=7.4 Hz), 1.70-1.94 (4H, m), 2.15 (1H, d, *J*=5.1 Hz), 4.73-4.80 (1H, m), 6.42 (1H, s), 7.32-7.51 (10H, m). ^{13}C NMR δ 21.05, 70.9, 83.1, 102.0, 115.4 (q, *J*=7.4 Hz), 121.7, 125.7, 128.5, 129.5, 131.7, 137.4, 137.6. ^{19}F NMR δ –64.83 (s).

(*Z*)-6,6,6-Trifluoro-2,2-dimethyl-4-(2-phenylethynyl)hex-4-en-3-ol (5f) and (*E*)-7-phenyl-2,2-dimethyl-4-(trifluoromethyl)hept-4-en-6-yn-3-ol (6f)

Combined yield 64%, R_f =0.50 (*n*-hexane:AcOEt=4:1). IR (neat) ν 3853, 3734, 3675, 3648, 3567, 3462, 3060, 3022, 2968, 2910, 2874, 2205, 1625, 1491, 1479, 1467, 1443, 1397, 1367, 1341, 1262, 1162, 1123, 1092, 1054, 1012, 937, 919, 881, 834, 756, 688, 643 cm^{-1}. Anal. Calcd for $C_{16}H_{17}F_3O$: C, 68.07; H, 6.07. Found: C, 68.33; H, 5.75.

5f ^{1}H NMR δ 1.07 (9H, s), 2.08 (1H, d, *J*=8.1 Hz), 4.45 (1H, d, *J*=8.1 Hz), 6.08 (1H, q, *J*=9.0 Hz), 7.26-7.47 (10H, m). ^{13}C NMR δ 9.6, 29.2, 70.2 (q, *J*=1.9 Hz), 84.2, 95.9 (q, *J*=1.2 Hz), 121.3 (q, *J*=273.6 Hz), 121.6, 122.9 (q, *J*=34.7 Hz), 128.4, 129.3, 131.9, 138.9 (q, *J*=5.6 Hz). ^{19}F NMR δ –57.13 (d, *J*=9.0 Hz).

6f ^{1}H NMR δ 1.08 (9H, s), 2.39 (1H, d, *J*=8.7 Hz), 4.63 (1H, d, *J*=8.4 Hz), 6.51 (1H, s), 7.26-7.47 (10H, m). ^{13}C NMR δ 10.4, 71.5, 82.8, 101.7 (q, *J*=1.3 Hz), 114.6 (q, *J*=7.5 Hz), 121.8, 128.5, 129.5, 131.6, 142.0 (q, *J*=27.3 Hz). ^{19}F NMR δ –64.72 (s).

2,5-Diphenyl-3-(2,2,2-trifluoroethyl)furan (7a) [2]

To a 30 mL two-necked flask containing THF (3 mL) at 0 °C were successively added 0.104 g (0.343 mmol) of a 70:30 mixture of **5a** and **6a** and 66 mg of $PdCl_2(PhCN)_2$ (0.017 mmol), and the whole solution was stirred for 24 h at room temperature. After the usual

work-up and chromatographic separation (*n*-hexane:cyclohexane=4:1) afforded 0.0529 g (0.175 mmol) of **7a**. Yield 51%, R_f =0.35 (*n*-hexane:cyclohexane=4:1). White crystal, mp 75-77 °C. ^{1}H NMR δ 3.47 (2H, q, *J*=10.5 Hz), 6.77 (1H, s), 7.25-7.75 (10H, m). ^{13}C NMR δ 31.3 (q, *J*=31.0 Hz), 108.7, 111.8 (q, *J*=3.1 Hz), 123.8, 125.9 (q, *J*=276.6 Hz), 126.5, 127.7, 128.1, 128.7, 128.8, 130.2, 130.3, 151.2, 153.0. ^{19}F NMR δ –66.37 (t, *J*=11.3 Hz).

References

1. Fujiwara, M.; Narizuka, S. 5,5,5-Trifluoro-3-penten-1-ynes and their preparation method. JP 2006-83066, March 30, 2006.
2. Qing, F.-L.; Gao, W.-Z.; Ying, J.-W. *J. Org. Chem.* **2000**, *65*, 2003–2006.
